# The Interplay between Adipose Tissue Properties and Levels of NT-proBNP in People with HIV

**DOI:** 10.1155/2023/6199388

**Published:** 2023-11-04

**Authors:** Mads-Holger Bang Jacobsen, Anne Marie Reimer Jensen, Andreas Dehlbæk Knudsen, Thomas Benfield, Ruth Frikke-Schmidt, Børge Nordestgaard, Shoaib Afzal, Klaus Fuglsang Kofoed, Marco Gelpi, Susanne Dam Nielsen

**Affiliations:** ^1^Viro-Immunology Research Unit, Department of Infectious Diseases 8632, Rigshospitalet, University of Copenhagen, Copenhagen, Denmark; ^2^Department of Cardiology, Herlev and Gentofte Hospital, Copenhagen University Hospital, Copenhagen, Denmark; ^3^Department of Cardiology, The Heart Center, Rigshospitalet, University of Copenhagen, Copenhagen, Denmark; ^4^Center of Research & Disruption of Infectious Diseases, Amager and Hvidovre Hospital, University of Copenhagen, Hvidovre, Denmark; ^5^Department of Clinical Medicine, Faculty of Health and Medical Sciences, University of Copenhagen, Copenhagen, Denmark; ^6^Department of Clinical Biochemistry, Rigshospitalet, University of Copenhagen, Copenhagen, Denmark; ^7^Department of Clinical Biochemistry, Copenhagen University Hospital, Herlev and Gentofte Hospital, Herlev, Denmark; ^8^Department of Radiology, Rigshospitalet, University of Copenhagen, Copenhagen, Denmark

## Abstract

**Objective:**

We aimed to assess the association between low N-terminal pro-brain natriuretic peptide (NT-proBNP) and body mass index (BMI), adipose tissue distribution, adiponectin, and HIV-specific risk factors among people with HIV (PWH).

**Methods:**

We included 811 PWH with measurement of height, weight and waist circumference, blood samples analyzed for NT-proBNP, and visceral-(VAT) and subcutaneous (SAT) adipose tissue areas measured from CT-scans. Low concentrations of NT-proBNP were defined as concentrations below the limit of quantification (5.9 pmol/L). Associations were explored with multivariable logistic regression analyses adjusted for relevant confounders.

**Results:**

We identified 471 (58%) individuals with low concentrations of NT-proBNP. Increasing BMI was associated with higher odds of low NT-proBNP (adjusted OR (aOR) 1.06 (95% CI: 1.01–1.11) per 1 kg/m^2^). Central obesity and large areas of VAT were associated with higher odds of low NT-proBNP (aOR 1.66 (1.16–2.36) and aOR 1.69 (1.09–2.62), respectively). Higher adiponectin was associated with lower odds of low NT-proBNP (aOR 0.86 (0.79–0.95) per 10% increase). No associations were found between low NT-proBNP and HIV-specific risk factors.

**Conclusions:**

In PWH, low NT-proBNP is associated with an adverse adipose tissue profile with high BMI, central obesity, accumulation of VAT, and low adiponectin.

## 1. Introduction

Cardiometabolic diseases are a main cause of non-AIDS-related morbidity and mortality in people with HIV (PWH) [1], and the risk of cardiovascular disease (CVD), including heart failure, is significantly higher among PWH than in the background population despite effective treatment with combination antiretroviral therapy (cART) [2]. Natriuretic peptides are secreted from cardiomyocytes in response to increases in intracardiac pressure and elicit their primary role as cardioprotective hormones through constriction of the efferent renal arterioles, inhibition of cardiac fibrosis, and suppression of the renin-angiotensin-aldosterone system (RAAS) [3]. Elevated levels of B-type natriuretic peptides (BNP) and the biologically inactive N-terminal prohormone of BNP (NT-proBNP) are associated with heart failure and mortality in both PWH and the general population [4–7], and current guidelines from both the European Society of Cardiology and the American Heart Association recommend measurement of BNP/NT-proBNP for both diagnosis and prognosis of heart failure [8, 9].

Interestingly, while the risk of heart failure and CVD is strongly associated with increasing body mass index (BMI), levels of natriuretic peptides are lower in obese individuals [10–12]. This paradox is known as “the natriuretic handicap” [13] and might in itself be associated with worse cardiovascular outcomes as insufficient levels of natriuretic peptides cause water retention, potentially promoting heart failure [3], and have been linked with poor cardiometabolic health [14].

Abdominal obesity and adipose tissue dysfunction are common among PWH [15, 16], and chronic inflammation is a hallmark of both untreated and treated HIV infection [17]. Previous studies have found higher concentrations of NT-proBNP in PWH compared with uninfected controls [7, 18, 19]. However, knowledge regarding the interplay between natriuretic peptides and HIV-associated changes to adipose tissue properties is sparse and limited to a single study reporting lower concentrations of BNP in PWH than controls when stratifying for BMI [20].

We aimed to investigate the association between concentrations of NT-proBNP and adipose tissue quantity, distribution, and function in a large cohort of well-treated PWH. Furthermore, we aimed to investigate potential HIV-related risk factors for low NT-proBNP. We hypothesized that low concentrations of plasma NT-proBNP were associated with (1) higher BMI, (2) central obesity, large VAT, and large subcutaneous adipose tissue (SAT) areas, and (3) lower concentrations of adiponectin.

## 2. Methods

### 2.1. Study Population

**Figure 1:**
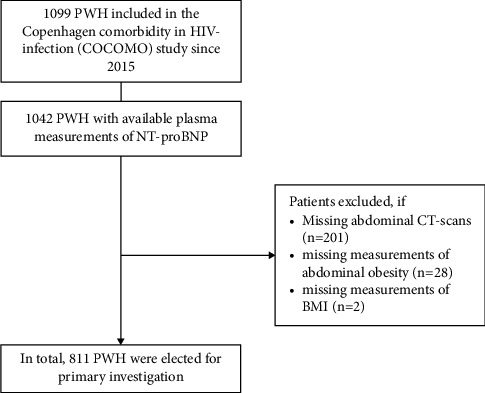
Flow chart of study inclusion. The figure illustrates the selection process by which the final study population was reached.

The Copenhagen Comorbidity in HIV infection (COCOMO) study is a prospective, observational study aiming to assess the burden and pathogenesis of comorbidity in PWH. The study includes 1,099 PWH enrolled between March 2015 and December 2016 [21]. All participants provided oral and written informed consent, and the study was approved by the Ethics Committee of the Capital Region of Denmark (H-8-2014-004) and the Danish Data Protection Agency (30–1454). The present analyses included all PWH with both available biobank samples for measurement of NT-proBNP and measures of adipose tissue properties (Figure 1).

### 2.2. Clinical Assessments

All participants underwent a physical examination with measurements of height, weight, waist circumference, and calculation of BMI performed by the trained medical staff [21]. Information on demographics, smoking, and use of medication was collected from questionnaires. Data regarding HIV infection, including cART, CD4^+^ cell counts, CD4^+^ nadir, date of HIV-diagnosis, and hepatitis C virus (HCV)-antibodies, were obtained from review of all participants' medical records.

### 2.3. Biochemical Analyses

NT-proBNP was measured in plasma collected at baseline and stored at −80°C [22]. Analyses were performed by sandwich electrochemiluminescence-immunoassay (ECLIA) at the Department of Clinical Biochemistry, Rigshospitalet. Analyses for LDL, glucose, creatinine, and adiponectin were performed at the Department of Clinical Biochemistry, Herlev Hospital [21]. The estimated glomerular filtration rate (eGFR) was calculated based on serum creatinine using the Chronic Kidney Disease Epidemiology Collaboration (CKD-EPI) equation [23].

### 2.4. Definitions

Low concentrations of NT-proBNP were defined as plasma concentrations below the limit of quantification (5.9 pmol/L). BMI was divided into categories in accordance with the World Health Organization's classification (underweight: BMI <18.5 kg/m^2^, normal weight: 18.5–24.9 kg/m^2^, overweight: 25.0–29.9 kg/m^2^, and obese: BMI ≥30 kg/m^2^) [24]. Central obesity was defined as a waist circumference >80 cm for women and >94 cm for men in accordance with the International Diabetes Federation [25]. Large VAT and SAT areas were ascribed PWH with areas in the upper quartile (≥141.1 cm^2^ and ≥184.8 cm^2^, respectively). Hypertension was defined as antihypertensive treatment and/or having ≥140 mmHg systolic and/or ≥90 mmHg diastolic blood pressure [26]. Dyslipidemia was defined as LDL ≥4.16 mmol/L (160 mg/dL) and/or lipid-lowering treatment [27]. Treatment with an antidiabetic drug for diabetes and/or nonfasting venous plasma glucose ≥11.1 mmol/L and/or HbA1c ≥6.5% was used to define diabetes. Exposure to lipotoxic cART was defined as ever exposed to either stavudine, zidovudine, and/or didanosine.

### 2.5. CT Scans

Thoracic and abdominal CT scans including contrast enhanced CT angiography were performed with a 320-multidetector scanner (Aquilion One ViSION Edition, Canon, Japan) in a single rotation (275 ms) at Rigshospitalet. Measurements of VAT and SAT from single-slice CT-scans at the level of the 4^th^ lumbar vertebra were performed by trained personnel using commercially available CT software (Fat Measurement, Aquilion ONE; Canon, Japan). VAT area was defined as adipose tissue delineated by the intra-abdominal muscular compartments, while SAT was defined as adipose tissue superficial to the abdominal muscles as done previously (Supplemental Figure 1) [16].

Left atrial volume was determined using axial views. The endocardial borders were manually traced on 15–20 tomographic slices, depending on the size of the atrial chamber. The left atrial appendage was included in the measurement, while the pulmonary veins were meticulously excluded from the assessment of the atrial cavity [28]. LA volume index (LAVi) was calculated by dividing LA volume with body surface area (BSA), which was calculated from the Du Bois formula as follows:

(1) $$\begin{array}{c} \left[\text{weight}{\left(\text{kg}\right)}^{0.425}\times \text{height}{\left(\text{cm}\right)}^{0.725}\right]\times 0.007184. \end{array}$$

### 2.6. Statistical Analyses

Comparisons of baseline characteristics between PWH with and without low NT-proBNP were performed with unpaired *t*-tests for normally distributed continuous variables or the Mann–Whitney *U*-test for non-normally distributed continuous variables, and *χ*^2^ test for categorical variables. Normally distributed continuous variables were reported with means and standard deviations (SD), while non-normally distributed variables were reported with medians and interquartile ranges (IQR). Categorical data were reported with frequency counts and percentage of subjects within each category.

NT-proBNP was regarded as a dichotomous variable (low concentrations: yes/no). Logistic regression models were used when exploring the association of different adipose tissue properties with low NT-proBNP and odds ratios (OR) were reported with 95% confidence intervals (CI). In addition to a base model adjusted for age and sex, a multiple regression model was applied with additional adjustment for possible confounders including smoking, diabetes, hypertension, eGFR, and HCV-antibodies. These variables were chosen a priori based upon previously reported associations [8–10, 18]. Models with VAT, SAT, or adiponectin as exposure were also adjusted for BMI. A sensitivity analysis was performed with additional adjustment for LAVi. Furthermore, multiple linear regression models were applied as a sensitivity analysis to utilize the full range of NT-proBNP concentrations. NT-proBNP was log-transformed, and linear models were adjusted for the same potential confounders as the logistic models.

Multiple logistic regression models adjusted for age, sex, smoking, diabetes, hypertension, eGFR, HCV-antibodies, and BMI were used to explore possible associations between NT-proBNP and HIV-specific variables including time living with HIV, current CD4^+^ count, nadir CD4^+^ count (<200/*µ*L: yes/no), and exposure to lipotoxic cART (yes/no) tested one at a time.

A two-sided *p* value <0.05 was considered statistically significant. All analyses were performed using R version 4.1.2 (R Foundation for Statistical Computing, Vienna, Austria).

## 3. Results

### 3.1. Baseline Characteristics

We included 811 PWH from the COCOMO study with measurement of NT-proBNP, abdominal CT scans, and anthropometric measurements available (Figure 1). Of these, 672 (83%) also had a measurement of plasma adiponectin. We identified 471 (58%) with low concentrations of NT-proBNP. PWH with low NT-proBNP were younger and more frequently male than PWH without low NT-proBNP (Table 1). Overall, PWH with low NT-proBNP were less likely to have dyslipidemia, hypertension, and diabetes but had a higher mean BMI. PWH with low NT-proBNP were diagnosed with HIV more recently, mode of transmission was more frequently men who have sex with men, and they were more likely to be virally suppressed than PWH without low NT-proBNP.

### 3.2. Body Mass Index

**Figure 2:**
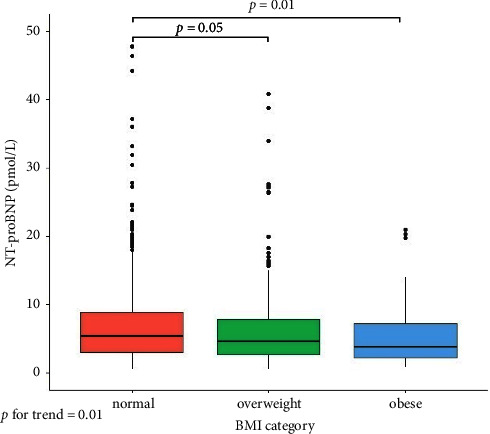
Concentrations of NT-proBNP in PWH according to BMI-category (normal 18.5–24.9 kg/m^2^, overweight 25.0–29.9 kg/m^2^, and obese ≥30 kg/m^2^). Statistical significance was determined using the Wilcoxon test (*p* < 0.05). BMI, body mass index; NT-proBNP, N-terminal prohormone of B-type natriuretic peptide.

Concentrations of NT-proBNP decreased with increasing BMI category, and overweight or obese PWH had lower NT-proBNP than those who had normal BMI (Figure 2). Increasing BMI was associated with 6% higher odds of low NT-proBNP per 1 kg/m^2^ increase in BMI, both when adjusting for sex and age (OR 1.06 [95% CI: 1.02–1.10], *p*=0.004) and after further adjustment for smoking, diabetes, hypertension, eGFR, and HCV antibodies (aOR 1.06 [1.01–1.11], *p*=0.009) (Table 2). Furthermore, these findings were consistent after additional adjustment for LAVi (Table 2) and when using linear regression models (Supplemental Table 1).

### 3.3. Adipose Tissue Distribution

Central obesity was associated with higher odds of low NT-proBNP in logistic regression models adjusted for sex and age (OR 1.52 [1.10–2.10], *p*=0.01) and with further adjustment for smoking, diabetes, hypertension, eGFR, HCV antibodies, and BMI (aOR 1.66 [1.16–2.36], *p*=0.005) (Table 2). Furthermore, these findings were consistent after additional adjustment for LAVi (Table 2) and when using linear regression models (Supplemental Table 1).

PWH with large VAT areas had higher odds of low NT-proBNP when adjusting for age, sex, smoking, diabetes, hypertension, eGFR, HCV antibodies, and BMI (aOR 1.69 [1.09–2.62], *p*=0.02), but the association was attenuated when further adjusting for LAVi (aOR 1.58 [0.95–2.62], *p*=0.08) (Table 2). Large SAT areas were not associated with low NT-proBNP. Similar findings were observed using linear regression models (Supplemental Table 1).

### 3.4. Adipose Tissue Function

Higher concentrations of adiponectin were associated with lower odds of low NT-proBNP in both the base models (OR 0.87 [0.80–0.96] per 10% increase in adiponectin, *p*=0.004) and after additional adjustments (aOR 0.86 [0.79–0.95] per 10% increase in adiponectin, *p*=0.003) (Table 2) and similar after further adjustment for LAVi (Table 2). Consistently, higher adiponectin was associated with higher NT-proBNP in linear regression models (Supplemental Table 1).

No associations were found between low concentrations of NT-proBNP and time since HIV diagnosis, current CD4^+^ count, nadir CD4^+^ count, or previous exposure to lipotoxic cART (data not shown).

## 4. Discussion

We investigated the association between natriuretic peptides and adipose tissue properties in a large cohort of well-treated PWH and found that low concentrations of NT-proBNP were associated with (1) higher BMI, (2) central obesity and large VAT areas, and (3) lower concentrations of adiponectin. We found no association between low NT-proBNP and large SAT areas or any of the HIV-specific variables tested.

Natriuretic peptides act upon multiple target organs and serve as regulators of cardiometabolic homeostasis by stimulating lipolysis, increasing GFR, and inhibiting both RAAS and cardiac fibrosis [3]. While high concentrations of BNP/NT-proBNP are used in diagnosis and prognosis of heart failure, low concentrations are also associated with a clinically important increased risk of adverse outcomes. Thus, genetically lower concentrations of natriuretic peptides have been linked with an adverse cardiometabolic profile with increased prevalence of metabolic syndrome, hypertension, and myocardial infarction [14]. Furthermore, patients with heart failure but concentrations of natriuretic peptides lower-than-expected may be at an increased risk of delayed diagnosis [29].

In the present study, we found that increasing BMI was associated with lower concentrations of natriuretic peptides which is in line with previous studies from the general population [10–12]. A previous study (*N* = 30) has also linked BMI and natriuretic peptides in PWH [20]. When stratified by both HIV-status and BMI, authors reported the lowest concentrations of BNP among obese individuals with HIV which was ascribed to the accumulation of dysfunctional fat seen among PWH. Notably, the study was conducted under strict physiological conditions during RAAS-activation and only included 20 highly selected PWH. Although we did not include a control group in our study, we provide evidence for the first time of an inverse relationship between obesity and NT-proBNP in a large and unselected cohort of PWH and verify that the association between BMI and low natriuretic peptides in PWH is consistent after adjustment for potential confounders.

Several hypotheses have been proposed to explain this “natriuretic handicap” and potential mechanisms include decreased synthesis and release of natriuretic peptides from cardiomyocytes in obese individuals either due to downregulation of the natriuretic peptide system [30], hyperinsulinemia [31], or accumulation of epi- and myocardial fat leading to myocardial damage [32]. Hyperinsulinemia may especially contribute to natriuretic deficiency in PWH, as previous studies from the COCOMO cohort found that PWH are at increased risk of diabetes and that HIV-specific risk factors including time with HIV and prior immunodeficiency are associated with higher insulin resistance [33, 34]. A previous study in PWH also found an inverse correlation between BNP and HOMA-IR, but BNP remained lower in PWH than controls after adjusting for HOMA-IR suggesting that lower BNP in PWH cannot be attributed to hyperinsulinemia alone [20].

Although BMI is a useful indicator of overall adiposity, it is a crude measure of obesity and does not account for body composition or adipose tissue distribution. Previous research indicates that adipose tissue in different compartments differentially influence the association with natriuretic peptides, and that VAT is more strongly associated with lower concentrations of natriuretic peptides than SAT [35–37]. PWH are at increased risk of abdominal obesity [15] as well as redistribution of fat from the subcutaneous to the visceral compartment [38] and might therefore be at particular high risk of low NT-proBNP. Interestingly, we found specifically central obesity and VAT, but not SAT, to be associated with low concentrations of NT-proBNP.

The association between VAT and low concentrations of natriuretic peptides may be explained by increased clearance of BNP at the adipose tissue level due to an increased gene expression of NPR-C in VAT compared with SAT [39]. However, since NT-proBNP is not eliminated by the NPR-C receptor [40], the association between VAT and low NT-proBNP cannot only be explained by this mechanism. Interestingly, one study found that the association between VAT and NT-proBNP was attenuated after additional adjustment for insulin resistance [35], suggesting that hyperinsulinemia may also facilitate natriuretic deficiency in individuals with excess VAT.

Adiponectin is an adipokine that reduces inflammation and improves insulin sensitivity, and low concentrations are associated with increased risk of CVD [41]. Higher concentrations of adiponectin have previously been associated with higher natriuretic peptides in both healthy men [42] and patients with heart failure [43], and concentrations of adiponectin have been demonstrated to increase after intravenous administration of natriuretic peptides in patients with heart failure [44]. VAT might be the common link between the two hormones, as VAT is associated with both lower concentrations of natriuretic peptides and produces TNF-*α* that reduces adiponectin expression [45]. Thus, VAT accumulation in PWH may lead to lower concentrations of both NT-proBNP and adiponectin – especially among those previously exposed to lipotoxic cART, as lipotoxic cART may have long lasting detrimental effects upon levels of adiponectin [16]. In the present study, increase in concentrations of adiponectin was associated with a decreased risk of low NT-proBNP. However, we did not observe an association between exposure to lipotoxic cART and low concentrations of NT-proBNP.

Major strengths of the study include the large sample size, a well-characterized study population, and CT-measured VAT and SAT areas. However, as this was a cross-sectional, observational study, we cannot make conclusions on causality or statements about the temporal relationship between obesity and concentrations of NT-proBNP. We also cannot assess if the effect of obesity on NT-proBNP levels is more pronounced in PWH compared with the general population as we did not include a control group for this study. Another limitation was that 58% of PWH had NT-proBNP values below the limit of quantification, which limited our ability to reliably distinguish low concentrations of NT-proBNP from very low concentrations. In addition, our population consisted mainly of Caucasian males, which disallowed sex-stratified analyses, and more than 98% were on stable cART. Therefore, our findings may not be generalizable to other HIV-populations. Moreover, the definition of lipotoxic cART was narrow. However, we limited the definition to treatments with well-documented effects on adipose tissue physiology as done previously [16]. Finally, even when adjusting analyses for age, sex, and several comorbidities, additional unmeasured confounders may be present. However, similar findings in sensitivity analyses with additional adjustment for LAVi were reassuring.

## 5. Conclusion

The present study found that in PWH, low NT-proBNP is associated with an adverse adipose tissue profile with high BMI, central obesity, accumulation of VAT, and low adiponectin. Adequate levels of natriuretic peptides help regulate cardiovascular homeostasis by protecting against volume overload, hypertension, and structural heart disease, and our findings add another potential link between metabolic dysfunction and CVD in PWH.

Further studies comparing natriuretic peptides and adipose tissue properties in both PWH and controls are warranted to investigate the clinical impact of natriuretic peptide deficiency among PWH. In addition, a lower cut-point for BNP/NT-proBNP may be relevant when diagnosing acute heart failure in obese PWH who may be at particular high risk of both natriuretic peptide deficiency and heart failure and may present with lower-than-expected levels of natriuretic peptides.

## Figures and Tables

**Table 1:** Baseline characteristics for PWH with and without low concentrations of NT-proBNP^a^.

Characteristics	Total (*N* = 811)	Low NT-proBNP (*N* = 471)	Without low NT-proBNP(*N* = 340)	*p* value
Age, years, median [IQR]	50.5 [43.0–59.0]	47.4 [40.8–53.8]	53.9 [47.8–63.9]	<0.001
Sex, male, *n* (%)	701 (86.4)	425 (90.2)	276 (81.2)	<0.001
BMI, kg/m^2^, mean (SD)	25.0 ± 3.9	25.2 ± 3.8	24.6 ± 4.0	0.03
BMI category (WHO), *n* (%)				0.13
Underweight	21 (2.6)	10 (2.1)	11 (3.2)	
Normal	435 (53.6)	239 (50.7)	196 (57.6)	
Overweight	277 (34.2)	172 (36.5)	105 (30.9)	
Obese	78 (9.6)	50 (10.6)	28 (8.2)	
Central obesity, *n* (%)	434 (53.5)	245 (52.0)	189 (55.6)	0.32
VAT area, cm^2^, median [IQR]	80.9 [37.1–141.1]	77.0 [36.8–139.2]	87.9 [38.5–142.1]	0.27
SAT area, cm^2^, median [IQR]	127.7 [81.2–184.8]	131.6 [82.8–191.1]	123.9 [76.7–177.1]	0.15
Adiponectin, *μ*g/ml, median [IQR]	11.7 [8.4–15.6]	11.0 [8.2–14.1]	13.0 [9.0–18.9]	<0.001
Smoking, *n* (%)				0.052
Current	224 (28.2)	119 (25.7)	105 (31.6)	
Previous	295 (37.1)	166 (35.9)	129 (38.9)	
Never	276 (34.7)	178 (38.4)	98 (29.5)	
Dyslipidemia, *n* (%)	172 (22.1)	84 (18.6)	88 (27.0)	0.006
Lipid lowering treatment, *n* (%)	105 (13.7)	43 (9.5)	62 (19.6)	<0.001
Hypertension, *n* (%)	335 (43.3)	176 (38.8)	159 (49.8)	0.002
Antihypertensive treatment, *n* (%)	134 (17.7)	56 (12.6)	78 (24.9)	<0.001
Diabetes, *n* (%)	33 (4.2)	12 (2.6)	21 (6.4)	0.03
Antidiabetic treatment, *n* (%)	26 (3.2)	11 (2.3)	15 (4.4)	0.10
eGFR, mL/min, mean (SD)	88.6 ± 15.5	91.6 ± 14.4	84.5 ± 16.1	<0.001
Mode of transmission, *n* (%)				<0.001
MSM	582 (72.5)	363 (77.9)	219 (65.0)	
Intravenous drug user	11 (1.4)	2 (0.4)	9 (2.7)	
Heterosexual	159 (19.8)	77 (16.5)	82 (24.3)	
HIV duration, years, mean (SD)	14.4 ± 9.0	12.9 ± 8.7	16.6 ± 9.0	<0.001
Current CD4^+^ (cells/*μ*L), mean (SD)	724 ± 288	734 ± 289	710 ± 288	0.24
CD4^+^ nadir <200 (cells/*μ*L), *n* (%)	193 (23.8)	104 (22.1)	89 (26.2)	0.18
HIV RNA >50 (copies/mL), *n* (%)	27 (3.3)	10 (2.1)	17 (5.0)	0.02
Exposure to lipotoxic cART, *n* (%)	415 (51.2)	213 (45.2)	202 (59.4)	<0.001
HCV-positive antibodies, *n* (%)	81 (10.3)	44 (9.7)	37 (11.2)	0.73
Current cART, *n* (%)	799 (98.5)	464 (98.9)	335 (98.5)	1
Duration of cART, years, mean (SD)	11.0 ± 6.6	9.9 ± 6.5	12.5 ± 6.4	<0.001

NT-proBNP, N-terminal prohormone of B-type natriuretic peptide; IQR, interquartile range; BMI, body mass index; SD, standard deviation; HIV, human immunodeficiency virus; VAT, visceral adipose tissue; SAT, subcutaneous adipose tissue; MSM, men who have sex with men; RNA, ribonucleic acid; cART, combination antiretroviral therapy; HCV, hepatitis C virus. ^a^Dyslipidemia was defined as LDL ≥4.16 mmol/L (160 mg/dL) and/or lipid-lowering treatment. Hypertension was defined as antihypertensive treatment and/or having ≥140 mmHg systolic and/or ≥90 mmHg diastolic blood pressure. Treatment with an antidiabetic drug for diabetes and/or nonfasting venous plasma glucose ≥11.1 mmol/L and/or HbA1c ≥6.5% was used to define diabetes. Exposure to lipotoxic cART was defined as ever exposed to either stavudine, zidovudine, and/or didanosine. If numbers and percentages do not add up, it is due to missing values.

**Table 2:** Associations of adipose tissue measurements with low concentrations of NT-proBNP.

Adipose tissue measurement	Base model		Adjusted model		Adjusted for LAVi	
	OR [95% CI]	*p* value	OR [95% CI]	*p* value	OR [95% CI]	*p* value
BMI, per 1 kg/m^2^ increase	1.06 [1.02; 1.10]	0.004	1.06 [1.01; 1.11]	0.009	1.07 [1.01; 1.14]	0.02
Central obesity	1.52 [1.10; 2.10]	0.01	1.66 [1.16; 2.36]	0.005	1.72 [1.14; 2.60]	0.01
Large VAT area	1.38 [0.92; 2.06]	0.12	1.69 [1.09; 2.62]	0.02	1.58 [0.95; 2.62]	0.08
Large SAT area	1.09 [0.69; 1.71]	0.71	1.24 [0.76; 2.01]	0.39	1.07 [0.60; 1.90]	0.82
Adiponectin, per 10% increase	0.87 [0.80; 0.96]	0.004	0.86 [0.79; 0.95]	0.003	0.86 [0.76; 0.96]	0.008

BMI, body mass index; VAT, visceral adipose tissue; SAT, subcutaneous adipose tissue; LAVi, left atrial volume index. Values shown are odds ratios (95% CI) for having low concentrations of NT-proBNP adjusted for age and sex (base model) and after further adjustment for diabetes, hypertension, eGFR, HCV-antibodies (adjusted model) and LAVi. Additionally, models exploring associations between VAT, SAT, and adiponectin and low NT-proBNP were adjusted for BMI. Large VAT and SAT areas were ascribed PWH with areas in the upper quartile (≥141.1 cm^2^ and ≥184.8 cm^2^, respectively). 684 PWH were included in the adjusted model exploring associations between low NT-proBNP and BMI, central obesity as well as large VAT and SAT areas (538 when further adjusting for LAVi), while 593 were included in the adjusted model exploring associations between low NT-proBNP and adiponectin (467 when further adjusting for LAVi).

## Data Availability

According to Danish legislation and approvals from the ethical committee and data protection agency, the data underlying this article cannot be shared publicly. The data can be accessed at our institution upon reasonable request to the corresponding author.

## Additional Points

*Study Important Questions*. *What is already known about this subject?* Despite being associated with increased risk for cardiovascular disease, obesity is associated with low concentrations of natriuretic peptides. Abdominal obesity and adipose tissue dysfunction are prevalent among people with HIV (PWH), but knowledge about the association between natriuretic peptides and obesity is missing in this setting. *What are the new findings in your manuscript?* Low NT-proBNP is associated with an adverse adipose tissue profile with high BMI, central obesity, accumulation of VAT, and low adiponectin among PWH. *How might your results change the direction of research or the focus of clinical practice?* A lower cut-point for BNP/NT-proBNP may be relevant when diagnosing acute heart failure in obese PWH who may be at particular high risk of both natriuretic peptide deficiency and heart failure and may present with lower-than-expected levels of natriuretic peptides.

## Supplementary Materials

Supplementary Materials The supplementary material file contains: Supplemental Table 1: linear regression model showing the degree of change of log-transformed concentrations of NT-proBNP with regard to each adipose tissue measurement. Supplemental Figure 1: determination of VAT and SAT from CT-scans.
