# Supplementary material for: The Interplay between Adipose Tissue Properties and Levels of NT-proBNP in People with HIV
Source: J Obes. 2023 Nov 4;2023:6199388. doi: 10.1155/2023/6199388 (PMC10640655; doi:10.1155/2023/6199388)
Supplement: Supplementary Materials — The supplementary material file contains: Supplemental Table 1: linear regression model showing the degree of change of log-transformed concentrations of NT-proBNP with regard to each adipose tissue measurement. Supplemental Figure 1: determination of VAT and SAT from CT-scans. [file 6199388.f1.docx]

**SUPPLEMENTAL MATERIALS**

**The interplay between adipose tissue properties and levels of NT-proBNP in people with HIV Adipose tissue properties and NT-proBNP in HIV**

Mads-Holger B. Jacobsen^1^, Anne Marie Reimer Jensen^1,2^, Andreas D. Knudsen^1,3^, Thomas Benfield^4,5^, Ruth Frikke-Schmidt^5,6^, Børge G. Nordestgaard^5,7^, Shoaib Afzal^7^, Klaus F Kofoed^3,8^, Marco Gelpi^1^, Susanne D. Nielsen^1,5^

1. Viro-immunology Research Unit, Department of Infectious Diseases 8632, Rigshospitalet, University of Copenhagen, Copenhagen, Denmark.

2. Department of Cardiology, Herlev and Gentofte Hospital, Copenhagen University Hospital, Copenhagen, Denmark

3. Department of Cardiology, The Heart Center, Rigshospitalet, University of Copenhagen, Copenhagen, Denmark.

4. Center of Research & Disruption of Infectious Diseases, Amager and Hvidovre Hospital, University of Copenhagen, Hvidovre, Denmark.

5. Department of Clinical Medicine, Faculty of Health and Medical Sciences, University of Copenhagen, Copenhagen, Denmark.

6. Department of Clinical Biochemistry, Rigshospitalet, University of Copenhagen, Copenhagen, Denmark.

7. Department of Clinical Biochemistry, Copenhagen University Hospital, Herlev and Gentofte Hospital, Herlev, Denmark

8. Department of Radiology, Rigshospitalet, University of Copenhagen, Copenhagen, Denmark.

Content:

- **Supplemental Table 1.** Linear regression model showing the degree of change of log-transformed concentrations of NT-proBNP with regard to each adipose tissue measurement
- **Supplemental Figure 1.** Determination of VAT and SAT from CT-scans.

**Supplemental Table 1.** Linear regression model showing the degree of change of log-transformed concentrations of NT-proBNP with regard to each adipose tissue measurement

| **Adipose tissue measurement** | **Base model** | | **Adjusted model** | |
| --- | --- | --- | --- | --- |
|  | **Crude β-coefficient [95% CI]** | **p-value** | **Adjusted β-coefficient [95% CI]** | **p-value** |
| BMI, per 1 kg/m^2^ increase | -0.03 [-0.05; -0.02] | <0.001 | -0.03 [-0.04; -0.01] | <0.001 |
| Central obesity | -0.16 [-0.28; -0.04] | 0.01 | -0.13 [-0.25; <0.00] | 0.04 |
| Large VAT area | -0.12 [-0.27; 0.03] | 0.12 | -0.16 [-0.32; -0.01] | 0.04 |
| Large SAT area | 0.10 [-0.07; 0.27] | 0.24 | 0.10 [-0.07; 0.27] | 0.26 |
| Adiponectin, per 10% increase | 0.07 [0.04; 0.10] | <0.001 | 0.06 [0.03; 0.10] | <0.001 |

*Abbreviations: BMI, body mass index; VAT, visceral adipose tissue; SAT, subcutaneous adipose tissue.*

Values shown are β-coefficients (95% CI) representing the change in log-transformed concentrations of NT-proBNP. The base model was adjusted for age and sex while additional adjustments for diabetes, hypertension, eGFR and HCV-antibodies were performed in the adjusted model. Additionally, models exploring associations between VAT, SAT, adiponectin and NT-proBNP were adjusted for BMI. Large VAT -and SAT areas were ascribed PWH with areas in the upper quartile (≥ 141.1 cm2 and ≥ 184.8 cm2, respectively).

**Supplemental Figure 1.** Determination of VAT and SAT from CT-scans.


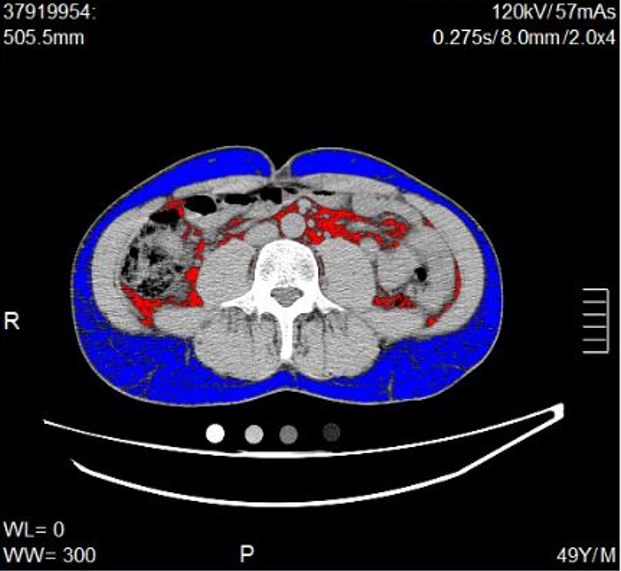


VAT area was automatically calculated from the adipose tissue delineated by the intraabdominal muscular compartments (red), while SAT area was calculated from the adipose tissue superficial to the abdominal muscles (blue).
